# Miniaturized BAW Filter for Wide Band Application Based on High-Q Factor Active Inductor

**DOI:** 10.3390/mi16060616

**Published:** 2025-05-24

**Authors:** Zhencheng Xu, Jiabei Pan, Feng Gao, Weipeng Xuan, Hao Jin, Jikui Luo, Shurong Dong

**Affiliations:** 1 College of Information Science and Electronic Engineering, Zhejiang University, Hangzhou 310027, China; 2 ZJU-Hangzhou Global Scientific and Technological Innovation Center, Hangzhou 311200, China; 3 College of Electronics & Information, Hangzhou Dianzi University, Hangzhou 310061, China

**Keywords:** bulk acoustic wave device, BAW filter, active inductor

## Abstract

BAW filters have been widely used in RF circuits, and their combination with integrated passive inductors is one of the most common forms of BAW filters. However, the large size of passive inductors increases the area of the filter, making it unable to meet packaging requirements. At the same time, their low quality factor (Q) severely degrades the performance of the BAW filter. This paper presents a miniaturized wide band BAW filter with small-size high-Q active inductor. The active inductor is implemented by a circuit topology with three common-source amplifiers constructed with N-type transistors. The three-stage topology uses a small-size transistor in the middle stage to reduce the parasitic capacitance at the input node, achieving a large inductive bandwidth. The simulation results show that the active inductor has variable inductance from 1 nH to 10 nH, and a quality factor of up to 4 K from 2 to 7 GHz. The 30 × 30 μm^2^ active inductor is embedded in a 4.55–5.05 GHz BAW filter ladder so as to substantially decrease filter size. Simulation results indicate that the BAW filter based on the active inductor achieves a low insertion loss of −1.1 dB, out-of-band rejection of −35 dB on the left side, and out-of-band rejection of −53 dB on the right side. Compared to the traditional passive inductor, this active inductor significantly improves the performance of the BAW filter while occupying a much smaller chip size of 0.83 × 0.75 mm^2^.

## 1. Introduction

RF filters, as an essential component in RF front-end modules, are widely used in wireless communication systems [1]. However, with the development of modern wireless communication systems such as 5G/6G and Wi-Fi 6E/7, the demand for RF filters with higher frequency and wider bandwidth is continuously growing [2,3]. Surface acoustic wave (SAW) filters [4] are unable to meet requirements above 3 GHz, and the bulk acoustic wave (BAW) resonator has become an indispensable component of 3–10 GHz wireless communication applications [5,6]. Furthermore, for ultra-wide bandwidth applications [7,8], the BAW filter needs to be combined with integrated passive inductors as a hybrid structure. The passive inductors can provide a wide bandwidth and out-of-band suppression zero point, while the BAW can provide a steep transition band [9,10]. For example, in Wi-Fi 6E/7 RF front-end modules, the BAW filter needs to be integrated with 3–4 passive inductors to meet the requirements of about 2 GHz bandwidth from 5.15 GHz to 7.125 GHz and a sideband suppression of around 2.4 GHz [10,11,12]. However, these large-sized passive inductors often make it difficult to meet the requirements of Wi-Fi 6E/7 RF filter packaging, such as the 1411 (14 mm × 11 mm) package size [13,14,15]. Additionally, the integration of 3–4 passive inductors can introduce mutual inductance, which decreases the quality factor (Q) and significantly reduces filter performance [16,17].

Advanced BAW resonators [18], such as ScAlN devices, offer enhanced electroacoustic coupling ($k_{t}^{2}$) and, thus, increase filter bandwidth. For example, AlN doped with 30% Sc can achieve around 20% $k_{t}^{2}$ [19,20,21]. However, achieving values exceeding 25% remains challenging, and the corresponding fractional bandwidth still typically stays around 10%. Moreover, high Sc doping often leads to higher fabrication costs and a reduced Q factor. In practice, this approach alone still faces difficulty in achieving bandwidths beyond 500 MHz near 5G operating frequencies.

In this work, a new method is proposed to address this issue by replacing passive inductors with active inductors, offering a promising alternative [22,23]. Active inductors have a very high Q factor, no mutual inductance, and high integration, which significantly reduces the size of BAW filters while maintaining their high performance. Unfortunately, the inductance of existing active inductors varies with frequency, which limits their application in BAW filters [22,23].

In this work, we propose an innovative active inductor and creatively integrate it into the BAW filter. The active inductor achieves a wide operating frequency range of 2–7 GHz and a high Q factor. A BAW filter based on this active inductor is developed to achieve a 500 MHz bandwidth.

## 2. Active Inductor Design

### 2.1. Description of Proposed Active Inductor Topology

**Figure 1:**
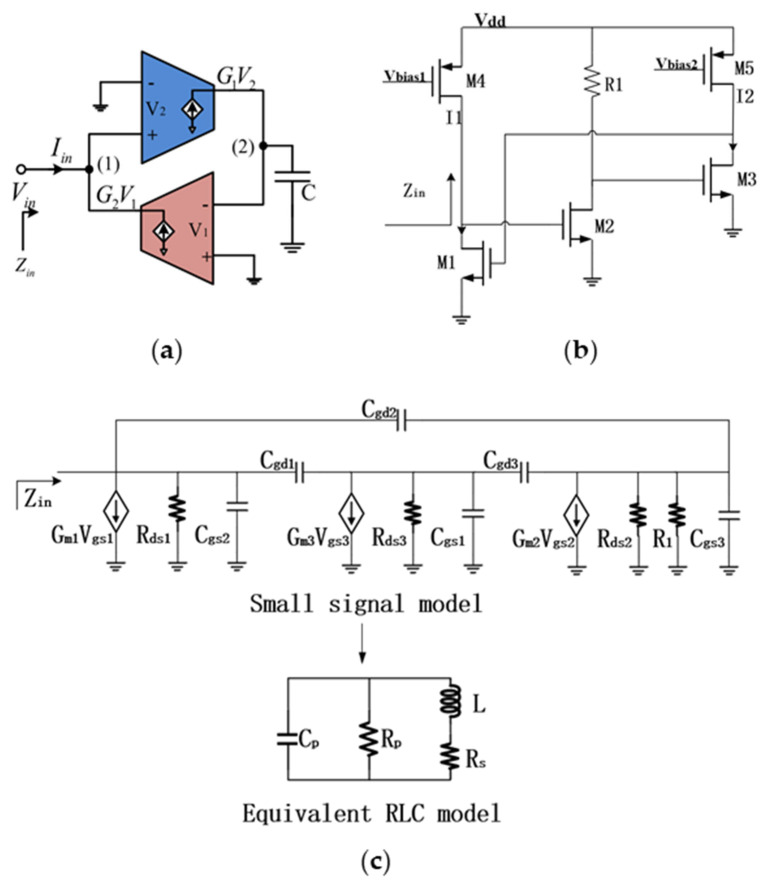
Schematic of active inductor: (**a**) gyrator-C topology; (**b**) proposed active inductor; (**c**) small signal model and equivalent RLC model.

The basic principle of the active inductor proposed in this article is to use a gyrator to convert capacitive impedance into inductive impedance [15]. As shown in Figure 1a, the input impedance of the circuit can be written as follows:

(1) $$Z_{in}=\frac{sC}{G_{1}G_{2}}=sL.$$

The proposed active inductor is shown in Figure 1b, and it consists of three NMOS transistors: M1, M2, and M3. Transistors M1 and M3 are biased by current sources M4 and M5, respectively, while M2 is connected to resistor R1. The common-source amplifier composed of M1 functions as the positive transconductance amplifier, while the common-source amplifiers composed of M2 and M3 transistors serve as the negative transconductance amplifier. Transistors M2 and M3 form a two-stage amplification structure, which not only enhances the gain and reduces inductance, but also disperses the capacitance from the input port to the ground. This increases its self-resonant frequency, enabling the active inductor to operate in a higher frequency band. The topology of this active inductor has a notable feature in that both the positive and negative transconductance amplifiers are composed of NMOS transistors fabricated using TSMC (Hsinchu, Taiwan) 180 nm CMOS process, while the two PMOS transistors solely serve as current sources. This design not only reduces circuit size, but also achieves higher gain.

In order to optimize the active inductor for high performance, we developed a small signal model. Figure 1c represents the small signal model of the active inductor and equivalent RLC model. In Figure 1c, $C_{gs}$, $C_{gd}$, and $G_{m}$ are the gate-source capacitance, gate-drain capacitance, and transconductance of the transistors, respectively.

Through the small signal model in Figure 1c, the input admittance of the active inductor can be represented as follows [15]:

(2) $$Y_{in}\approx sC_{gs2}+g_{m2}+\frac{1}{r_{ds1}}+\frac{g_{m1}(sC_{gd1}+g_{m2}g_{m3}(r_{ds2}//R1))-g_{m2}(g_{m3}+\frac{1}{r_{ds2//R1}})}{sC_{gs1}+sC_{gs3}}.$$

It is not difficult to obtain the various parameters of the RLC model of the active inductor from the input admittance expression [15] of the small signal model in Figure 1c. They can be derived as follows:

(3) $$\begin{array}{c} C_{P}\approx C_{gs2},\;R_{P}\approx \frac{r_{ds1}}{g_{m2}r_{ds1}+1},\\ R_{S}\approx {|S}^{2}L^{2}|\frac{g_{m1}C_{gd1}}{C_{gs1}+C_{gs3}}=\frac{\omega^{2}L^{2}g_{m1}C_{gd1}}{C_{gs1}+C_{gs3}},\\ L\approx \frac{C_{gs1}+C_{gs3}}{g_{m1}(g_{m2}g_{m3}(r_{ds2}//R1))-g_{m2}(g_{m3}+\frac{1}{r_{ds2}//R1})}. \end{array}$$

Considering the influence of frequency on the quality factor of the inductor, the quality factor of the inductor can be expressed as follows:

(4) $$Q(\omega )=\frac{-imag(Y_{in})}{real(Y_{in})}=\frac{\frac{g_{m1}g_{m2}g_{m3}(r_{ds2}//R1)-g_{m2}(g_{m3}+\frac{1}{r_{ds2//R1}})}{\omega {(C}_{gs1}+C_{gs3})}-\omega C_{gs2}}{g_{m2}+\frac{1}{r_{ds1}}+\frac{g_{m1}C_{gd1}}{C_{gs1}+C_{gs3}}}.$$

By using the formula of the equivalent RLC model, the self-resonant frequency of the inductor can be expressed as follows:

(5) $$SRF=\frac{1}{\sqrt{LC_{P}}}\approx \frac{1}{\sqrt{\frac{C_{gs2}(C_{gs1}+C_{gs3})}{\left(g_{m1})(g_{m2}g_{m3}(r_{ds2}//R1))-g_{m2}(g_{m3}+\frac{1}{r_{ds2}//R1}\right)}}}.$$

The above formulas can somewhat reveal the characteristics of the active inductor, particularly the influence of various circuit parameters on some key performance metrics of the proposed active inductor. It should be noted that the L listed in Formula (3) refers to the inductance in the parallel circuit model in Figure 1c, which is not exactly equivalent to the inductance in the series model. However, if the Q factor of the inductor is sufficiently high, its equivalent inductance is similar in both series and parallel models, thereby simplifying the subsequent analysis. From Formula (3), it can be observed that the inductance L is approximately positively correlated with the transistor transconductance and negatively correlated with the parasitic capacitance. The wide bandwidth of the proposed active inductor benefits from the low capacitance of the small-sized M2 transistor, while the gain is compensated through two-stage amplification. Formula (4) shows a positive correlation between the Q factor and transistor transconductance. When the transconductance ($g_{m2}$ and $g_{m3}$) of the M2 and M3 transistors in the circuit of Figure 1b becomes excessively large, the Q factor represented by Formula (4) may become negative because the real part of the impedance of the inductor is negative. Carefully setting circuit parameters can make the real part of the impedance of the inductor approach zero, which can result in a considerable Q factor.

### 2.2. Improvement of Q Factor and Inductance Stability

**Figure 2:**
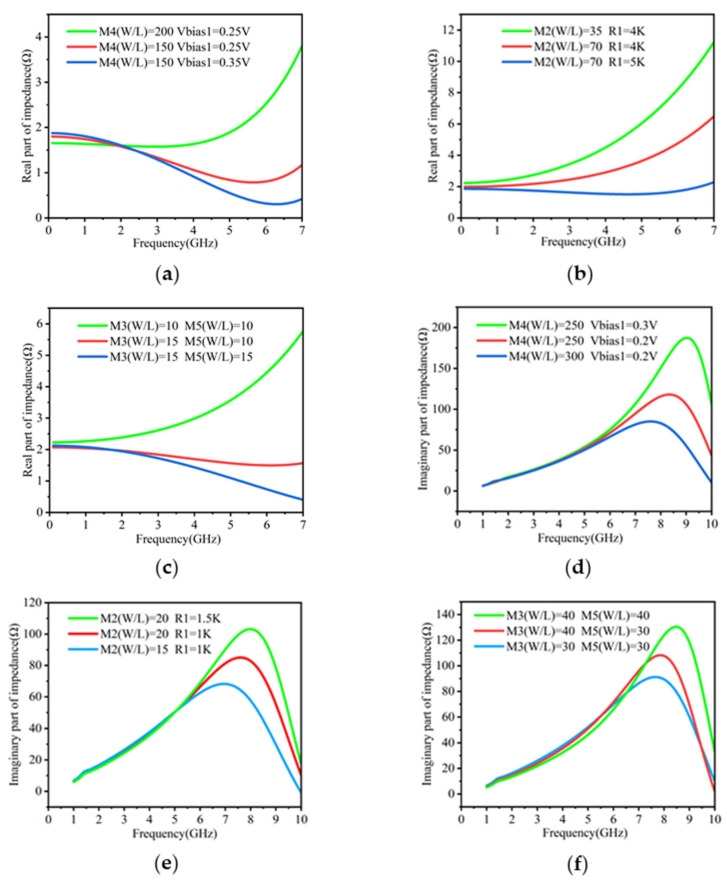
Relationship between impedance and parameters of active inductors: (**a**) influence of M4 aspect ratio and Vbias1 on real parts; (**b**) influence of M2 aspect ratio and R1 on real parts; (**c**) influence of M3 and M5 aspect ratios on real parts; (**d**) influence of M4 aspect ratio and Vbias1 on imaginary parts; (**e**) influence of M2 aspect ratio and R1 on imaginary parts; (**f**) influence of M3 and M5 aspect ratios on imaginary parts.

The proposed active inductor exhibits a high Q factor, which underscores its advantage. To achieve this, the real part of its impedance must be minimized. Several methods can be employed to reduce the real part of the impedance for the circuit shown in Figure 1b. Firstly, increasing the aspect ratio of M4 or decreasing Vbias1 can increase the bias current flowing through M1 and M4, which can reduce the real part of the impedance at low frequencies (e.g., 1 GHz), and the result is shown in Figure 2a. Secondly, as illustrated in Figure 2b, increasing R1 or the aspect ratio of M2 can reduce the real part over entire frequencies (1–7 GHz)—this is due to the decrease in $R_{S}$ in Formula (3). Finally, increasing the aspect ratio of M3 and M5 helps to reduce the real part at high frequencies (above 6 GHz) in Figure 2c—this is because the increase in the transconductance of M3 and M5 increases the self-resonant frequency of the active inductor according to Formula (5), thereby extending the inductor’s behavior towards higher frequencies (above 6 GHz).

Through extensive testing, it has been found that the real part of the impedance of the active inductor typically decreases with frequency, reaching a minimum point, after which it increases sharply. By carefully tuning the design, this minimum can be positioned close to zero, yielding a high Q factor. Furthermore, the minimum point can be shifted to target high Q factors at different frequency ranges.

The proposed active inductor also demonstrates satisfactory inductance stability, and its inductance changes slowly with frequency. To achieve this, the imaginary part of the impedance needs to exhibit a nearly linear relationship with frequency. There are several methods to linearize the imaginary part. Firstly, Figure 2d indicates that increasing the aspect ratio of M4 or decreasing Vbias1 helps to linearize the imaginary part. Secondly, reducing R1 or the aspect ratio of M2 also contributes to a more linear frequency dependence of the imaginary part, as depicted in Figure 2e. Thirdly, reducing the aspect ratios of transistors M3 or M5 further enhances the inductance stability, as demonstrated in Figure 2f.

Improving the Q factor of the active inductor while maintaining its inductance stability is sometimes contradictory. Therefore, it is necessary to make a tradeoff among multiple parameters.

### 2.3. Inductor Simulation Results

**Figure 3:**
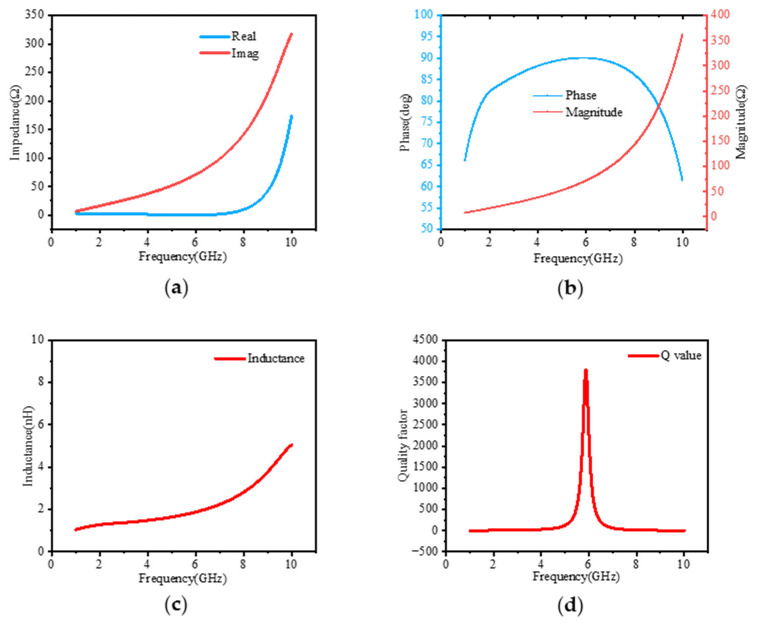
Simulation results of active inductor: (**a**) real and imaginary parts of impedance; (**b**) phase and magnitude; (**c**) inductance; (**d**) quality factor.

The active inductor was designed and simulated at the transistor level using the Cadence Virtuoso (version IC618 (Cadence Design Systems, San Jose, CA, USA)). The Cadence Spectre simulator was employed to evaluate its small-signal inductance, Q factor, and broadband behavior. This approach provides a reliable foundation for evaluating the active inductor’s integration into the BAW filter architecture. Figure 3 shows the simulation results of the active inductor, including the impedance, inductance, and Q factor of the active inductor.

The simulation results indicate that the active inductor operates from 2 to 7 GHz, only exhibiting an inductance variation of approximately 0.5 nH up to 6 GHz. This stability provides a significant advantage over other active inductors [22]. Additionally, the peak quality factor of this active inductor is close to 4K, substantially higher than that of other active inductors, which enables it to improve the performance of BAW filters [23].

**Figure 4:**
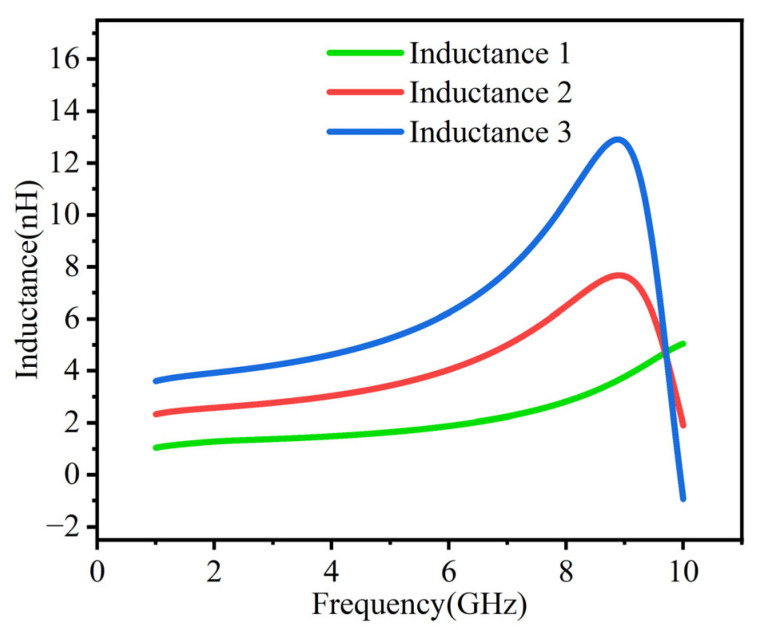
Comparison of different inductance levels.

Figure 4 demonstrates the tunability of the active inductor’s inductance, indicating that the inductor can be adjusted to between 1 nH and 6 nH around 4 GHz. Further testing revealed that the inductance range extends from 1 nH to 10 nH, effectively meeting the inductance requirements for BAW filters. Figure 4 shows that the inductance tends to increase beyond 7 GHz, which can actually be optimized in future designs. In this case, the inductance remains basically unchanged over a large frequency range, but there may be a loss of quality. Table 1 shows a comparison between the proposed active inductor and other active inductors. It can be seen that the proposed active inductor has a larger frequency range, better inductance stability, and a higher Q factor.

### 2.4. Inductor Layout

**Figure 5:**
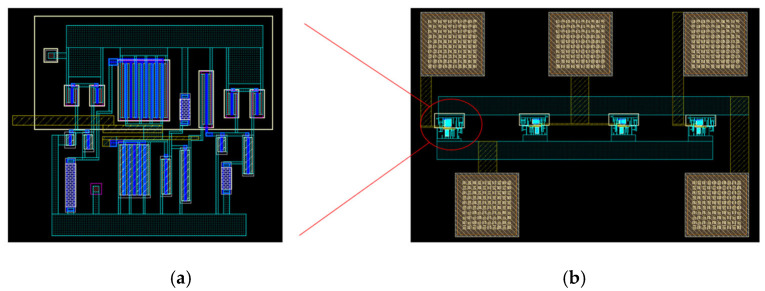
Layout of active inductors: (**a**) layout of single active inductor; (**b**) layout of active inductor array.

Figure 5 shows the layout design of the active inductor. Figure 5a shows a separate active inductor layout, occupying an area of 30 × 30 μm^2^. Figure 5b shows an array layout consisting of three active inductors and pads (the middle inductor is composed of two active inductor cells connected in parallel), occupying an area of 300 × 250 μm^2^.

## 3. BAW Filter with Active Inductor Embedded

### 3.1. Characteristics of BAW Resonator

**Figure 6:**
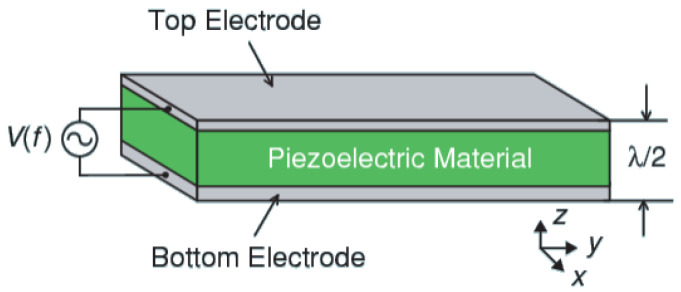
Structure of BAW resonator.

The bulk acoustic wave (BAW) resonator, as illustrated in Figure 6, is a layered structure consisting of two metal electrodes sandwiching a piezoelectric material. It serves as the core component of a BAW filter. The resonator operates based on the piezoelectric effect: when subjected to an external electric field, the piezoelectric layer deforms and consequently generates a secondary electric field. These interactions result in impedance variations that depend on frequency.

In this design, the resonator utilizes molybdenum (Mo) as both the top and bottom electrodes. The bottom electrode has a thickness of 107 nm, while the top electrode’s thickness ranges from 104 nm to 165 nm, depending on the specific resonator configuration. Sandwiched between the electrodes is a 300 nm thick Al_0.904_Sc_0.096_N piezoelectric film, which provides an electromechanical coupling coefficient ($k_{t}^{2}$) of approximately 9%. This material and structural configuration ensures effective acoustic confinement, impedance matching, and high-frequency performance.

**Figure 7:**
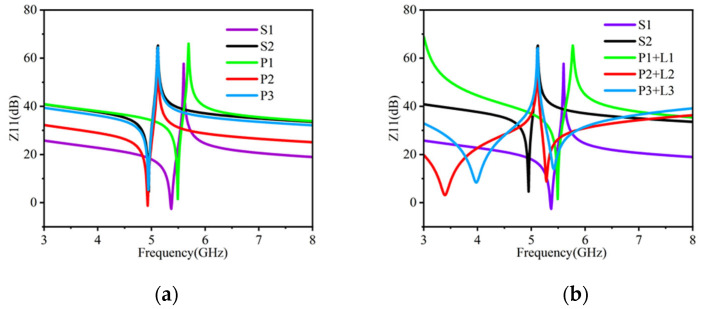
Impedance of BAW resonator: (**a**) single BAW resonator; (**b**) BAW resonator and inductor.

**Figure 8:**
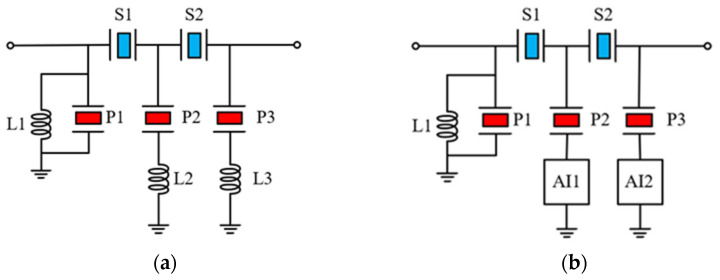
Schematic of BAW filters: (**a**) original BAW filter; (**b**) BAW filter after replacement of active inductors.

Figure 7 presents the impedance characteristics of five individual BAW resonators used in the filter, along with the impedance variations observed when the resonators are connected with an inductor. These configurations correspond to the BAW filter structure depicted in Figure 8.

### 3.2. Topology of BAW Filter and Its Combination with Active Inductor

In traditional ladder bandpass filters, the bandwidth is largely constrained by the electroacoustic coupling coefficient ($k_{t}^{2}$), making it challenging to achieve a wide bandwidth. In this work, the principle of band-stop is applied to the design of filters. In contrast to ladder bandpass filters, this can transform the original passband into a stopband and the original stopband into a passband, significantly improving bandwidth. The schematic of the proposed BAW filter is shown in Figure 8a, consisting of three parallel resonators and two series resonators. Its topology can be considered as a transposition of a ladder bandpass filter, but we still need to carefully consider the arrangement of resonators to achieve optimal performance. As shown in Figure 8a, the inductors L2 and L3 are connected in series with resonators P2 and P3, which can generate zero points on both sides of the passband and cause out-of-band rejection. However, the out-of-band rejection remains suboptimal. Therefore, inductor L1 is connected in parallel with resonator P1 to further enhance the out-of-band rejection, because L1 and P1 can form another zero point on the right side of the passband. In addition, further optimization is necessary to reduce insertion loss and flatten the passband.

**Figure 9:**
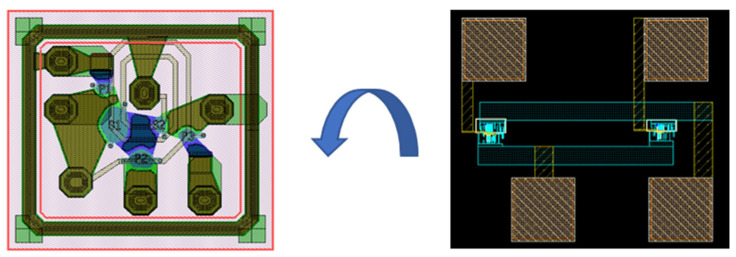
Layout of BAW filter and active inductor.

Figure 8b illustrates the topology of the reconstructed BAW filter using active inductors, where inductors L2 and L3, originally connected in series with resonators P2 and P3, have been replaced by active inductors (AI). Specifically, the replacement is achieved by directly connecting one terminal of resonator P2 and P3 to the Zin port of the active inductor in Figure 1b. Unlike passive inductors, active inductors have an intrinsic bias current, and an external signal is injected into them through the port to display the characteristics of the inductor, but it cannot affect the bias of the circuit itself. Normally, we can use capacitors to connect the active inductor with the required device. Since BAW resonators exhibit capacitive impedance outside of two resonant frequencies, they can be directly connected to the active inductor without affecting the normal operation of the BAW filter. In Figure 8a, the quality factor of inductors L2 and L3 has a critical impact on the insertion loss of the BAW filter, which is also one of reasons for replacing them with high Q active inductors. Figure 9 shows the layout of the BAW filter and the active inductor array that is bonded to it. The proposed compact BAW filter with active inductors only occupies 0.83 × 0.75 mm^2^.

### 3.3. Influence of Q Factor and Inductance Stability on BAW Filter

**Figure 10:**
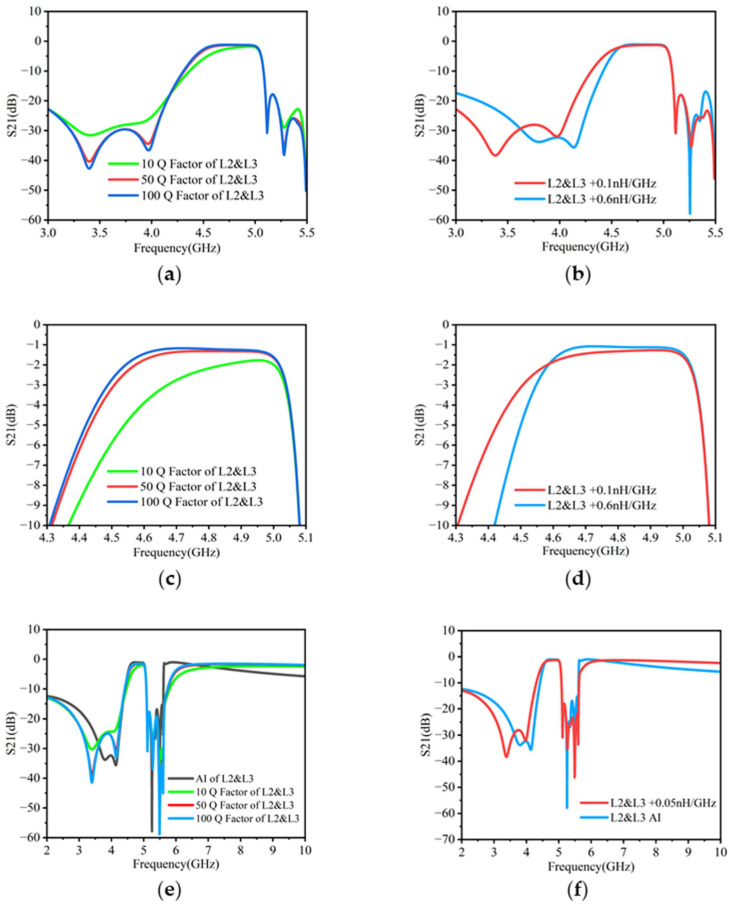
Influence of Q factor and inductance stability on BAW filter: (**a**) influence of Q factor on BAW filter; (**b**) influence of inductance stability on BAW filter; (**c**) influence of Q factor on BAW filter passband; (**d**) influence of inductance stability on BAW filter passband; (**e**) influence of Q factor of passive and active inductors on filters over wider frequency range; (**f**) influence of inductance stability of passive and active inductors on filters over wider frequency range.

The Q factor and inductance stability of inductors L2 and L3 have some impact on the performance of the BAW filter shown in Figure 8. These effects are further illustrated in Figure 10. As seen in Figure 10a, an increase in the Q factor results in a reduction in the insertion loss of the BAW filter and an enhancement of its out-of-band rejection. However, when the Q factor is 10 or lower, the overall performance of the BAW filter deteriorates. Figure 10b shows the impact of inductance stability on filter performance. It is observed that the fluctuation of inductance has a complex and detrimental impact on BAW filters, including a reduction in bandwidth and a weakening of out-of-band rejection. This introduces additional challenges to the design of BAW filters. To further evaluate broadband performance, the simulation frequency range has been extended up to 10 GHz in Figure 10e,f. The results show that the filter response using active inductors remains comparable to that of passive inductors within and beyond the intended operating band (4.55–5.05 GHz), confirming the robustness of the proposed approach.

It is worth noting that the current filter architecture lacks a low-pass stage, which results in limited suppression at higher frequencies (above 7 GHz). This is a potential direction for future enhancement.

As previously discussed, the proposed active inductor maintains both a high Q factor and stable inductive behavior within the target frequency range. Beyond approximately 7 GHz, however, its impedance no longer increases with frequency—instead, it gradually decreases. This deviation from ideal inductive behavior is mainly attributed to reduced transistor gain and the growing influence of parasitic capacitances at higher frequencies, which undermine the effectiveness of the Gm-C inductance emulation. Despite this limitation, the active inductor performs effectively within the operational range of the filter, validating its suitability for compact, integrated BAW filter applications.

### 3.4. Simulation Results and Comparison

**Figure 11:**
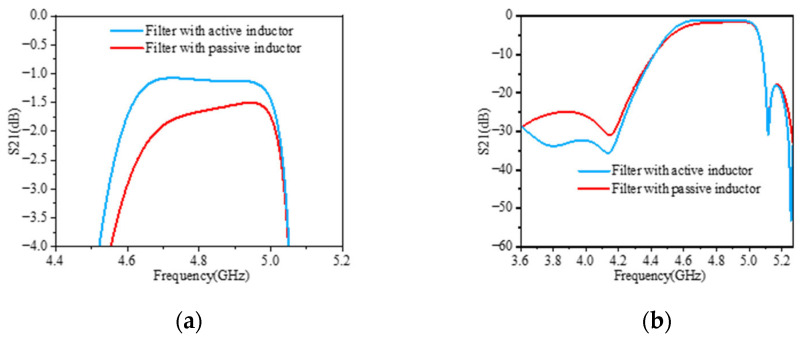
Simulation results of two BAW filters: (**a**) comparison results of insertion loss within passband of filter after using active inductors; (**b**) comparison results of S21 of BAW filter after using active inductors.

The BAW resonators were initially modeled using the Mason equivalent circuit, implemented in Keysight ADS (version 2023 Update 2 (Keysight Technologies, Santa Rosa, CA, USA)) for frequency-domain analysis. Simulations of the BAW filter embedded with active inductors were then conducted using RFIC Dynamic Link, enabling seamless co-simulation between ADS and Cadence. This methodology preserves the high-frequency physical behavior of the BAW devices while allowing accurate transistor-level simulation of the active inductor. Figure 11 presents the simulation results of the BAW filter before and after using active inductors. In Figure 11a it can be seen that, compared to typical passive inductors with a Q factor of 50, using active inductors reduces the insertion loss in the passband of the BAW filter by about 0.5 dB. Figure 11b demonstrates that the reconstructed BAW filter utilizing active inductors achieves an out-of-band rejection of −35 dB on the left side of the passband, which is 11 dB higher than that of the original filter. The highest out-of-band rejection on the right side of the passband reaches −53 dB, which is 19 dB higher than that of the original filter. These improvements are attributed to the extremely high Q factor of active inductors, which also causes faster attenuation of the filter at the passband edge. Another advantage of active inductors is their compact size, as the space occupied by two active inductors does not exceed 2000 μm^2^, which allows the filter to easily meet packaging requirements even when more inductors are configured. A comparison with other BAW filters proposed during previous research is provided in Table 2.

## 4. Conclusions

In this work, we designed a miniaturized 500 MHz bandwidth BAW filter and optimized it by embedding high-Q active inductors, which led to a reduction in insertion loss and an improvement in out-of-band rejection. The proposed active inductor achieves a tunable inductance from 1 to 10 nH, and a quality factor of up to 4K from 2 to 7 GHz. Its improved inductance stability also enables more effective combination with the BAW filter. Based on the principle of band-stop BAW filters, we configured multiple BAW resonators with different impedance characteristics to create a wide band filter topology, then we integrated the active inductor into the BAW filter. The simulation results show that the insertion loss within the passband of the BAW filter after active inductor reconstruction is −1.1 dB, with a left out-of-band rejection of −35 dB and a maximum out-of-band rejection of −53 dB on the right side. Compared to passive inductors, the 4.55–5.05 GHz BAW filter with active inductors exhibits significant improvements in various performance aspects and occupies a much smaller chip size of 0.83 × 0.75 mm^2^. These results confirm the active inductor’s value in enhancing BAW filter performance for advanced wireless communication applications.

## Figures and Tables

**Table 1:** Comparison with other active inductors.

Ref.	Frequency (GHz)	Inductance (nH)	Inductance Stability	Quality Factor
[14]	0.5–4	8–20	0.6 nH per GHz	3–10
[16]	1.76–6.18	3–10	0.45 nH per GHz	0–400
[22]	0.1–3.8	1.55–4	0.88 nH per GHz	10–76
[23]	0.8–2.7	0–5	2.9 nH per GHz	0–50
This work	2–7	1–10	0.08 nH per GHz	100–4000

**Table 2:** Comparison with other BAW filters.

Ref.	Frequency (GHz)	Bandwidth (MHz)	Insertion Loss (dB)	Out-of-Band Rejection (dB)	Size (mm^2^)
[1] 2023 BAW	4.73–4.97	240	0.93	25	1.1 × 0.75
[5] 2022 BAW	2.97–3.29	324	2.2	10	0.6 × 0.4
[10] 2022 BAW+ passive inductor	2.02–2.14	120	2.3	40	2.5 × 1.5
This work BAW+ active inductor	4.55–5.05	500	1.1	35	0.83 × 0.75

## Data Availability

The data are included in the article.

## Abbreviations

The following abbreviations are used in this manuscript:

AI	Active inductor
BAW	Bulk acoustic wave
NMOS	N-channel Metal-Oxide-Semiconductor
PMOS	P-channel Metal-Oxide-Semiconductor
Q	Quality
RF	Radio Frequency
RLC	Resistor-Inductor-Capacitor
SAW	Surface acoustic wave
SRF	Self-resonant frequency

## References

[1] Ding R., Xuan W., Gao F., Cao T., Jin H., Luo J., Ma F., Dong S. (2023). Key High-Performance N79 Band AlScN BAW Resonator and Filter With the Consideration of Area Effect. IEEE Trans. Electron Devices.

[2] Ding R., Xuan W., Gao F., Jiang H., Wang W., Jin H., Luo J., Dong S. (2024). Compact and High Steep Skirts Hybrid Heterogeneous Integrated N77 Full Band BAW Filter Based on Band-Stop Theory. IEEE Electron Device Lett..

[3] Tilhac C., Razafimandimby S., Cathelin A., Bila S., Madrangeas V., Belot D. A tunable bandpass BAW-filter architecture using negative capacitance circuitry. Proceedings of the 2008 IEEE Radio Frequency Integrated Circuits Symposium.

[4] Yang Y., Dejous C., Hallil H. (2023). Trends and Applications of Surface and Bulk Acoustic Wave Devices: A Review. Micromachines.

[5] Lv L., Shuai Y., Bai X., Huang S., Zhu D., Wang Y., Zhao J., Luo W., Wu C., Zhang W. (2022). Wide Band BAW Filter Based on Single-Crystalline LiNbO_3_ Thin Film with Insulating Bragg Reflector. IEEE Trans. Ultrason. Ferroelectr. Freq. Control.

[6] Guerrero E., Gimenez A., Heeren W., Al-Joumayly M. BAW Filter Trends for 5G and Beyond. Proceedings of the 2024 IEEE International Microwave Filter Workshop (IMFW).

[7] Karnati K., Schaefer M., Yusuf W., Rothemund R., Al-Joumayly M., Fattinger G. 5G C-V2X Filter Using BAW Technology. Proceedings of the 2021 IEEE MTT-S International Microwave Filter Workshop (IMFW).

[8] AlJoumayly M., Rothemund R., Schaefer M., Heeren W. 5G BAW Technology: Challenges and Solutions. Proceedings of the 2022 IEEE 22nd Annual Wireless and Microwave Technology Conference (WAMICON).

[9] Aliouane S., Kouki A.B., Aigner R. RF-MEMS switchable inductors for tunable bandwidth BAW filters. Proceedings of the 5th International Conference on Design & Technology of Integrated Systems in Nanoscale Era.

[10] Yu H., Wang X., Peng X., Zhang L., Jiang P., Zhao X., Deng L., Ma J. Performance Optimization of FBAR Filters with Wafer-Level Chip-Scale Package Using Embedded Matching Inductors on Multilayer PCB. Proceedings of the 2022 IEEE MTT-S International Microwave Workshop Series on Advanced Materials and Processes for RF and THz Applications (IMWS-AMP).

[11] Cheon S.J., Bang D.H., Park J.Y. Miniaturization of FBAR duplexer using PCB embedded high Q spiral inductors. Proceedings of the 2009 European Microwave Conference (EuMC).

[12] Zhou C., Dou W., Qin R., Lu J., Yang Y., Mu Z., Yu W. (2024). Highly Doped Single Crystal Al_1-x_Sc_x_N Bulk Acoustic Resonators for High-Frequency and Wideband Applications. IEEE Trans. Electron Devices.

[13] Herbert T.B., Hyland J.S., Abdullah S., Wight J., Amaya R.E. (2021). An Active Bandpass Filter for LTE/WLAN Applications Using Robust Active Inductors in Gallium Nitride. IEEE Trans. Circuits Syst. II Express Briefs.

[14] Oraha J.A., Younis A.T. Design, Realization, and Simulation of CMOS Active Inductors for RF Applications. Proceedings of the 2022 2nd International Conference on Advances in Engineering Science and Technology (AEST).

[15] Momen H.G., Yazgi M., Kopru R., Saatlo A.N. CMOS high-performance UWB active inductor circuit. Proceedings of the 2016 12th Conference on Ph.D. Research in Microelectronics and Electronics (PRIME).

[16] Hammadi A.B., Mhiri M., Haddad F., Saad S., Besbes K. A 1.82–4.44 GHz reconfigurable bandpass filter based on tunable active inductor. Proceedings of the 2016 11th International Design & Test Symposium (IDT).

[17] Allidina K., Mirabbasi S. A widely tunable active RF filter topology. Proceedings of the 2006 IEEE International Symposium on Circuits and Systems.

[18] Lu H., Hao X., Yang L., Hou B., Zhang M., Wu M., Dong J., Ma X. (2025). Recent Advances in AlN-Based Acoustic Wave Resonators. Micromachines.

[19] Qian Y., Wang X., Zhang Y., Liu C., Zhu Y. Demonstration of 8-Inch Thin-Film Sc_0.3_A1_0.7_N BAW Resonator with High Electromechanical Coupling Coefficient. Proceedings of the 2024 IEEE MTT-S International Conference on Microwave Acoustics & Mechanics (IC-MAM).

[20] Gerbe R., Yang Y., Hou D. ScAlN BAW resonator technology with coupling coefficients up to 21% for high performance filter design. Proceedings of the 2024 IEEE Ultrasonics, Ferroelectrics, and Frequency Control Joint Symposium (UFFC-JS).

[21] Wang X., Liu C., Zhang Y., Yang W., Qian Y., Liu P., Quek Z.J., Hong Y., Yi E., Woo Z. Thin Sc_0.2_Al_0.8_N Film Based 15 GHz Wideband Filter: Towards mmWave Acoustic Filters. Proceedings of the 2023 International Electron Devices Meeting (IEDM).

[22] Lai Y.-L., Zheng C.-Y. (2011). Electromagnetic Characteristics of a Novel Radio-Frequency Complementary Metal–Oxide–Semiconductor Active Inductor. IEEE Trans. Magn..

[23] Belen A., Belen M.A., Palandöken M., Mahouti P., Tari Ö. (2023). Design and Realization of Broadband Active Inductor Based Band Pass Filter. Chin. J. Electron..

